# *luox*: novel validated open-access and open-source web platform for calculating and sharing physiologically relevant quantities for light and lighting

**DOI:** 10.12688/wellcomeopenres.16595.2

**Published:** 2021-06-03

**Authors:** Manuel Spitschan, James Mead, Chris Roos, Chris Lowis, Ben Griffiths, Paul Mucur, Michael Herf

**Affiliations:** 1 Department of Experimental Psychology, University of Oxford, Oxford, UK; 2 Sleep and Circadian Neuroscience Institute (SCNi), University of Oxford, Oxford, UK; 3 Centre for Chronobiology, Psychiatric Hospital of the University of Basel, Basel, Switzerland; 4 Transfaculty Research Platform Molecular and Cognitive Neurosciences, University of Basel, Basel, Switzerland; 5 Go Free Range Ltd., London, UK; 6 Ghost Cassette Ltd., London, UK; 7 f.lux software LLC, Los Angeles, USA

**Keywords:** chronobiology, sleep research, environmental psychology, CIE, International Commission on Illumination, light, ipRGCs, melanopsin, cones, rods, alpha-opic radiance, alpha-opic irradiance, equivalent daylight illuminance, equivalent daylight luminance, EDI, EDL, non-visual effects of light, spectrum, web platform, open access, open source

## Abstract

Light exposure has a profound impact on human physiology and behaviour. For example, light exposure at the wrong time can disrupt our circadian rhythms and acutely suppress the production of melatonin. In turn, appropriately timed light exposure can support circadian photoentrainment. Beginning with the discovery that melatonin production is acutely suppressed by bright light more than 40 years ago, understanding which aspects of light drive the 'non-visual' responses to light remains a highly active research area, with an important translational dimension and implications for "human-centric" or physiologically inspired architectural lighting design. In 2018, the International Commission on Illumination (CIE) standardised the spectral sensitivities for predicting the non-visual effects of a given spectrum of light with respect to the activation of the five photoreceptor classes in the human retina: the L, M and S cones, the rods, and the melanopsin-containing intrinsically photosensitive retinal ganglion cells (ipRGCs). Here, we described a novel, lean, user-friendly, open-access and open-source platform for calculating quantities related to light. The platform, called *luox*, enables researchers and research users in chronobiology, sleep research and adjacent field to turn spectral measurements into reportable quantities. The *luox* code base, released under the GPL-3.0 License, is modular and therefore extendable to other spectrum-derived quantities. *luox* has been endorsed by the CIE following black-box validation.

## Introduction

Light profoundly affects human physiology and behaviour ^1^. Exposure to light in the evening and at night can suppress the production of melatonin ^2– 6^ and delay phase of the circadian rhythm ^3, 7– 13^, while morning light exposure advances the circadian phase ^10– 12^. Additionally, exposure to light modulates alertness ^14– 16^, and there is emerging evidence for a direct role of light in regulating mood ^1, 17, 18^. These *‘non-visual effects’* of light are mediated by a subset of the retinal ganglion cells which express the photopigment melanopsin ^19– 24^, a short-wavelength sensitive pigment with a peak spectral sensitivity near 480 nm ^20, 25, 26^. Importantly, melanopsin provides a pathway for signalling environmental illumination that is independent of the ‘canonical’ photoreceptors in the retina, the cones, of which there are three types that differ in their spectral tuning (the, L, M and S cones), and the rods.

The modulation of non-visual physiology by melanopsin is supported by a set of key studies in the early 2000s led by Brainard and colleagues ^4, 5^ (data reanalysed in 27– 29), and by Skene and colleagues ^6^ (data reanalysed in 27) which determined the action spectrum for acute human melatonin suppression during night-time light exposure, showing a clear short-wavelength peak. Action spectrum data for circadian phase shifting is limited ^7, 30, 31^, not least due to the complexity in implementing protocols to assess light-induced circadian phase shifts, though existing data are consistent with a dominant role of melanopsin ^7, 29– 31^. Furthermore, melatonin suppression responses to bright evening light persist in patients without demonstrable cone and rod function ^32, 33^, clearly supporting the role of melanopsin in non-visual responses in retinal and ocular disease ^34^.

While many fundamental aspects of the non-visual effects of light have been characterised in experimental and field studies, there are many unknowns and understanding the effects of light on human physiology and behaviour remains a highly active area of investigation. Recent studies have investigated temporal integration properties of the human circadian system ^8, 35– 39^, and have exploited silent-substitution techniques or metameric lights to characterise the non-visual response to pairs of lights that differ only in melanopsin stimulation ^40, 41^ or S cone stimulation ^42^.

With many response characteristics still being under investigation, mechanistic insights about the non-visual effects of light exposure are now increasingly finding their way into real-world applications. A recent international consensus statement identified criterion light levels to minimise the detrimental effects of light at the wrong time and maximise the positive ones ^43^. At the same time, principled approaches to realising physiologically inspired lighting based on scientific evidence are emerging (e.g. see 44). Both the development of recommendations and applying basic neuroscience findings in architectural lighting design require the ability to use a common currency to quantitatively describe the effect of light on people.

Science is a cumulative effort that requires the aggregation of studies to facilitate meta-analytic efforts and evidence synthesis (see, e.g., 27 and 45 for recent efforts to aggregate data). To enable a clear interpretation of the results and “future-proof” ^46^ research efforts requires adequate documentation of study conditions going well beyond current standard practice. A recent examination of 71 studies on the biological effects of light revealed that 55% (39/71) did not report any information about spectrum, and an overwhelming majority (87%; 62/71) only reported light levels in lux ^47^, representing a quantity weighting L and M cone activity, which is inappropriate given the converging evidence of the dominant role of melanopsin ^27– 29, 48^. Furthermore, there is large variability in what is considered “dim light”, with light levels called “dim” in practice spanning almost two log units ^47^. Since a key biomarker for circadian phase, the “dim light melatonin onset” (DLMO) ^49, 50^, hinges upon data collection in dim lighting conditions.

Recommendations and guidelines for the measurement, recording and reporting of the lighting conditions have been made independently by Spitschan *et al.* ^51^ and Knoop *et al.* ^52^, with a more recent comprehensive guide, also encompassing study characteristics other than light, being published by the International Commission on Illumination (CIE) ^53^. However, while recommendations and guidelines are quite important, they are only one aspect, and to make them easy to be applied, tooling is often required. To support adoption of the recent CIE standard S 026 ^54^, the CIE recently released an Excel spreadsheet ^55^, requiring the proprietary Excel software. There are also a range of tools available for calculations to run locally on the user’s computer, such as the Python package colour, requiring a functional Python installation.

Here, we introduce a novel web-based platform called *luox* for calculating, reporting and sharing physiological quantities related to light. All calculations are performed in a modern browser and require no software installations on the user side.

## Methods

### Implementation

luox (RRID: SCR_020994) is implemented in JavaScript, HTML & CSS using React and chart.js. The source code is available under the GPL-3.0 License at GitHub ^56^. luox is deployed at https://luox.app/. Further details on implementation are given on the about page.

### Operation

**Figure 1.**
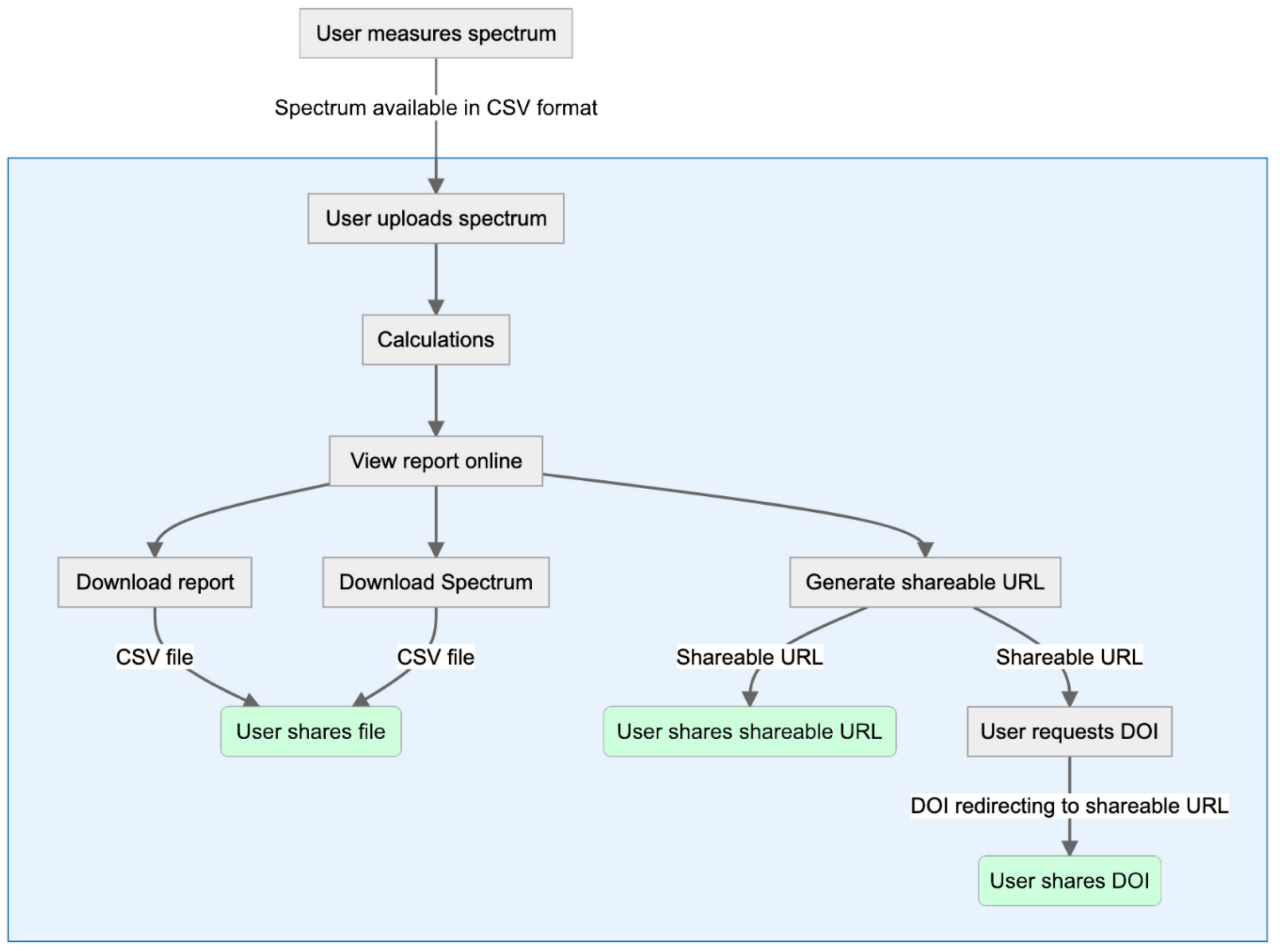
Workflow diagram.

**Figure 2.**
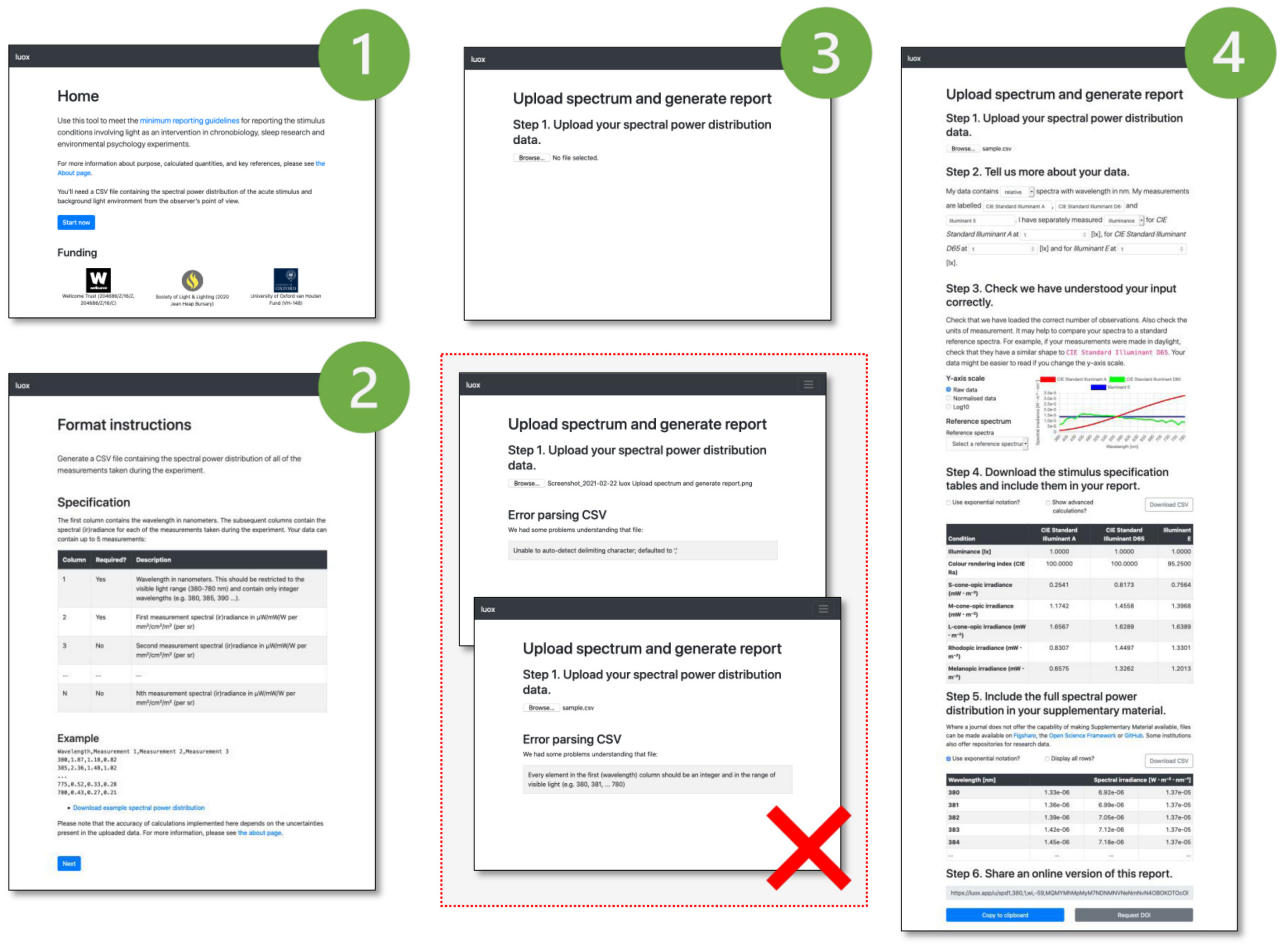
*luox* graphical user interface. ( **1**) Home and landing page; ( **2**) formatting instructions for spectra to be uploaded; ( **3**) upload dialogue; (X) failure of upload with indicative error messages; ( **4**) report with calculated quantities.

luox can be accessed online using any contemporary browser. The operational workflow is shown in Figure 1. The user measures an irradiance or radiance spectrum using their spectroradiometer and stores the spectrum in a CSV file. This file is then uploaded into the platform, which performs a series of calculations (detailed below) and visualises the spectrum in a report. This report can then be viewed in the browser, but also downloaded as a CSV file for sharing, e.g. as supplementary CSV file. The platform also allows for downloading the spectrum again as a CSV file. In addition, luox generates a shareable URL, which encodes the uploaded spectra in a URL (see below). A DOI can be requested which redirects to the shareable URL. The graphical user face is shown in Figure 2.

### Use case

The luox platform includes a wizard, which details the requirements for files and expected file formats, and also includes a sample file containing three CIE Illuminants in the F series in 5 nm spacing. Upon uploading the file, calculations are performed, giving access to the workflow described above and in Figure 1.

### Calculations

The platform implements as a set of photometric, colorimetric, colour rendition and α-opic calculations based on a user-supplied irradiance [W/m ^2^/nm] or radiance [W/m ^2^/sr/nm] spectral power distribution *S*( *λ*). It is assumed that the user has

*Photometry*. luox implements the following photometric calculations:

*•   Illuminance [lux] or luminance [cd/m ^2^]*. Illuminance (for irradiance spectra) and luminance (for radiance spectra) corresponds to the spectrum weighted by the photopic luminosity function, *V*( *λ*), and multiplied by the constant 683 lm/W. *V*( *λ*) is based on psychophysical measurements (for a review, see 57) and was first standardised by the CIE in 1924 ^58^ and forms the basis of current photometry ^59^.

*Colorimetry*. We implement the following colorimetric calculations:

*•   CIE 1931 xy chromaticity (2° observer).* The chromaticity coordinates are a way to identify the colour appearance of a spectrum. The CIE 1931 xy chromaticity diagram, also called the horseshoe, is based on the XYZ colour matching functions standardised by the CIE in 1931 ^60^ based on 2° colour matching experiments by Wright ^61^ and Guild ^62^. The chromaticity coordinates are calculated by weighting the spectrum by the $\bar{x}$ ( *λ*), $\bar{y}$ ( *λ*), and $\bar{z}$ ( *λ*) colour matching functions, yielding tristimulus coordinates *X*, *Y* and *Z* and then normalising: $x=\frac{X}{X+Y+Z}$ and $y=\frac{X}{X+Y+Z}$ . It is useful to note that CIE 1931 $\bar{y}$ ( *λ*) and CIE 1924 *V*( *λ*) are equivalent.

*•   CIE 1964 x _10_y _10_ chromaticity (10° observer).* The CIE has also standardised 10° colour matching functions $\bar{x}$ _10_( *λ*), $\bar{y}$ _10_( *λ*), and $\bar{z}$ _10_( *λ*), and associated chromaticity coordinates *x*_10_ and *x*_10_^63^. The 10° colour matching functions are based on psychophysical measurements done by Speranskaya ^64^ and Stiles and Burch ^65^.

*α-opic quantities following CIE S 026/E:2018.* In 2018, the International Commission on Illumination (CIE, abbreviating the French *Commission Internationale de l’Eclairage*) released the new International Standard CIE S 026/E:2018 (“CIE System for Metrology of Optical Radiation for ipRGC-Influenced Responses to Light”, ^54^), standardising the spectral sensitivities for ipRGC-mediated responses to light, and associated quantities. The spectral sensitivities of the L, M and S cones correspond to those developed by Stockman and colleagues ^66, 67^ and endorsed by the CIE ^68^. The rods’ spectral sensitivity corresponds to the standardised scotopic luminosity function, $V^{\prime }$ ( *λ*) ^59^, based on psychophysical measurements ^69^. The melanopsin spectral sensitivity curve in CIE S 026/E:2018 is the same as the one used in the influential Lucas *et al.* article ^70^ and associated Irradiance Toolbox (see supplement of 70), based on previous proposals ^71, 72^. To make calculations of CIE S 026/E:2018 related quantities accessible, the CIE released an Excel-spreadsheet based toolbox ^55^ and associated user guide ^73^.

*luox* implements the following quantities based on CIE S 026/E:2018:

*•   α-opic irradiance [mW/m ^2^] or radiance [mW/m ^2^/sr]*. The α-opic irradiance or radiance of a spectrum is the weighted sum of the spectrum and the α-opic spectral sensitivity. Here and in the definitions for EDI/EDL and ELR below, “α-opic” is a placeholder term that can be filled by any of the five photoreceptors, the L, M and S cones, the rods and melanopsin. For example, the spectral irradiance or radiance weighted by the L cone spectral sensitivity is called the L-cone-opic irradiance or radiance, and the spectral irradiance or radiance weighted by the melanopsin spectral sensitivity is called the L-cone-opic irradiance or radiance.

*•   α-opic equivalent daylight illuminance (EDI) and luminance (EDL).* The α-opic equivalent daylight illuminance (EDI) or luminance (EDL) calculates the photopic (il)luminance of a standard daylight spectrum (CIE Standard Illuminant D65, corresponding approximately to daylight with a correlated colour temperature of 6500K) that matches the α-opic (ir)radiance. Alternatively stated, the EDI/EDL tells us the (il)luminance that a daylight would have to have to yield the same α-opic (ir)radiance.

*•   α-opic efficacy of luminous radiation (ELR)*. The α-opic efficacy of luminous radiation is the ratio between the α-opic (ir)radiance and photopic (il)luminance, providing a simple, normalised indicator of the *α-opic* “content” of a spectrum. The melanopic ELR, i.e. the melanopic efficacy of luminous radiation, is similar to the the M/P ratio method proposed elsewhere ^74^.

In addition to linear notation, it is possible to toggle the display to exponential notation. While display of the calculated values is truncated to four decimal digits, the downloaded report, and indeed all underlying calculations, includes the numbers up to floating point precision (double-precision 64-bit binary format IEEE 754).

### Encoding of spectral power distributions

Spectral power distributions are typically stored as files in an MS Excel spreadsheet, comma-separated (CSV), XML (e.g. 75), JSON or other schema-based formats (e.g. 76). Storage of files requires an infrastructure, e.g., a server. To lose this requirement and enable the sharing of spectral power distribution data without sharing files, we (M.H.) developed a library with no external dependencies called *spdurl* written in JavaScript. *spdurl* encodes a spectral power distribution in a URL accurately and concisely. While the RFC for URLs (RFC 2616 ^77^) does not specify an upper length limit, many web browsers may truncate it to 2 kB, which we pragmatically adopt as the limit for URLs here.

spdurl exploits the following aspects of spectral distributions:

*•   Wavelength sampling:* Wavelengths are assumed to be uniformly spaced, allowing us to write only the first value and an increment, with the total number of samples implicit in the number of samples given. Some spectroradiometers produce nonuniform wavelength spacing, so we expect these users to resample to a uniform spacing, as with the rest of luox.

*•   Compression:* We compress the spectra using a scheme that encodes spectral bands in two URL-safe bytes each, across a variety of units. The measurement process within CCD spectrometers uses a shared shutter for all elements in the array, allowing us to share an exponent for all values. This is also often done in HDR formats, like Radiance RGBE ^78^. As a base for our shared exponent, we chose $\sqrt{\sqrt{2}}$ , sacrificing 1/8 bit to quantisation, rather than 1/2 bit if we had used 2 as a base. We do not constrain the exponent value (large exponents just use more bytes), so extremely small and large values can be represented, but most exponents use one or two bytes. In this way, energy can be stored as a linear value even at very small irradiances without worrying about range limits. While the best available CCD spectrometer arrays are specified to measure 16 linear bits, real-life signal to noise is much lower. We began with an 18-bit mantissa, but subsequently determined that for most uses, 12 bits of gamma-encoded data were sufficient for our calculations. To balance the accuracy of smaller and larger values, we informally determined that *γ* = 2.0 gave lower error than linear or cubic encodings. We perform rounding to 12 bits to avoid quantisation bias, which may result in small changes in the spectrum and derived quantities.

*•   String encoding:* We encode the resulting string as a URL-safe base64 (RFC 4648 ^79^), meaning that each 12-bit value can be written using two bytes, with no padding.

*•   Spectral resolution limits*: Since we use two bytes per value, this means that visible spectra can be encoded in a valid URL (2kB) down to about 0.5 nm spacing. Meters that have high spectral resolution may wish to resample to fewer values before encoding.

*•   Spectral units*: We use a dictionary of datatypes (30 to date), which can represent spectral quantities (so that “/nm” is common to all) using just 2-3 bytes. For instance, the shorthand “uwi” is used to represent “uW/cm ^2^/nm”. Additionally, action spectra, transmittance, radiance, and quantal units are available using similar short abbreviation given in the software. Many file formats assume the reader knows the units in use; in spdurl, we make no such assumption, and so, this field is required.

*•   Meta-data specification*: Measurement conditions, like time zone, date, location, and user-specified name are allowed as optional metadata, when there is space at the end.

As an example, our library can encode a spectral radiance distribution specified between 380 and 780 nm with 1 nm spacing using 804 bytes, and a 10 nm sample (36 bands) from an X-Rite meter can occupy only 90 bytes, as succinct as the following:

**spd1,380,10,wi,4,uJuIuI4m68488W_h-38t7c6S6J5A3i4M4G4G3N1u0Hx-w0v0uwuFtmr-qsp2ohncrBvsxz2j**

*spdurl*-encoded spectral power distributions can be shared across platforms, e.g., in *luox*:

https://luox.app/u/spd1,380,10,wi,4,uJuIuI4m68488W_h-38t7c6S6J5A3i4M4G4G3N1u0Hx-w0v0uwuFtmr-qsp2ohncrBvsxz2j,nMeasurement%201

and in *fluxometer*:

https://fluxometer.com/rainbow/#!id=data/spd1,380,10,uwi,4,uJuIuI4m68488W_h-38t7c6S6J5A3i4M4G4G3N1u0Hx-w0v0uwuFtmr-qsp2ohncrBvsxz2j

*spdurl* (RRID: SCR_020992) is written in JavaScript for use on the web and node.js servers. It is available under the MIT License (code base at Github, npm package here). *spdurl* can be used as a standalone package independent of *luox*, and we hope that it will be attractive to other users.

### Requesting DOIs

To facilitate the sharing of URLs encoded in *luox*, we offer the deposition of encoded URLs in the Oxford Research Archive. Users need to complete a form with minimal information, and upon manual approval, a DOI will be assigned.

### Reference spectra

luox allows for graphical comparisons of uploaded spectra with a series of CIE reference spectra. These include Standard Illuminant A, Standard Illuminant D65, and Illuminants C, D50, D75, F1 through F12, FL3.1 through 3.15, HP1 through HP5, LED-B1 through LED-B5, and LED-BH1, LED-RGB1, LED-V1, LED-V2 ^80^.

### Validation and CIE endorsement

*luox* has been validated by the CIE. The “black-box validation”, using 43 spectra from various sources (19 at 5 nm and 24 at 1 nm), confirmed that the calculations performed by *luox* was within defined tolerance intervals. The CIE report states ^81^:

“This software incorporates methods, formulae, spectral function calculations and spectra from the International Commission on Illumination (CIE). The CIE endorses this software having made a black-box evaluation of the software as of Feb. 11, 2021, finding that the software performs satisfactorily. This software is not a replacement for the CIE publications and works from which it is derived. The user is advised to consult the original publications and works for proper understanding of and calculation of the result of this software.”

## Discussion

Here, we present the *luox* platform for facilitating and sharing calculations of physiologically relevant quantities related to light and lighting. *luox* is open-access and open-source. *luox* is fully functional and modular, enabling the incorporation of other spectrally derived quantities in the future.

## Data availability

No data are associated with this article.

## Software availability

Software available from: https://luox.app/

Source code available from: https://github.com/luox-app/luox

Archive source code at time of publication: https://doi.org/10.5281/zenodo.4594093 ^56^

License: GPL-3.0
