# Supplementary material for: luox: validated reference open-access and open-source web platform for calculating and sharing physiologically relevant quantities for light and lighting
Source: Wellcome Open Res. 2022 Jun 27;6:69. Originally published 2021 Mar 29. [Version 3] doi: 10.12688/wellcomeopenres.16595.3 (PMC8095192; doi:10.12688/wellcomeopenres.16595.3)
Supplement: Peer review history

# Peer review: Reviewer response for version 3

**DOI:** 10.21956/wellcomeopenres.19747.r51248

**Published:** 2022-07-01

**Authors:** Timothy Brown

**Affiliations:** 1 Faculty of Biology Medicine and Health, University of Manchester, Manchester, UK

No further comments

Are the conclusions about the tool and its performance adequately supported by the findings presented in the article?

Partly

Is the rationale for developing the new software tool clearly explained?

Yes

Is the description of the software tool technically sound?

Yes

Are sufficient details of the code, methods and analysis (if applicable) provided to allow replication of the software development and its use by others?

Yes

Is sufficient information provided to allow interpretation of the expected output datasets and any results generated using the tool?

Yes

Reviewer Expertise:

Circadian biology, vision, electrophysiology

I confirm that I have read this submission and believe that I have an appropriate level of expertise to confirm that it is of an acceptable scientific standard.

# Peer review: Reviewer response for version 1

**DOI:** 10.21956/wellcomeopenres.18289.r43480

**Published:** 2021-04-30

**Authors:** John P. Hanifin, Benjamin Warfield

**Affiliations:** 1 Department of Neurology, Thomas Jefferson University, Philadelphia, PA, USA; 2 Light Research Program, Thomas Jefferson University, Philadelphia, PA, USA

This is a well-written report from an experienced group of researchers. Their report describes a free, open source, and open access platform to calculate quantities and metrics to describe the ability of optical radiation to stimulate each of the five (α-opic) photoreceptor types in the human eye. Each photoreceptor potentially can contribute, via the melanopsin-containing intrinsically-photosensitive retinal ganglion cells (ipRGCs), to neuroendocrine, neurobehavioral and circadian effects of light in humans. Alongside these values, the tool provides commonly used colorimetric calculations. The International Standard CIE S 026:2018 defines spectral sensitivity functions, quantities and metrics in units of α-opic quantities that are compliant with the International System of Units (SI) and currently recommended by the CIE to report values in research studies and lighting applications relevant to the physiological effects of light. A parsimonious exploration of three spectra was entered into the platform: a monochromatic light source, a polychromatic white LED light source and a polychromatic incandescent light source. The resulting data were identical to those provided by the CIE excel toolbox. We were very impressed with the platform's abilities and ease-of-use. Finally, having the data encoded into an HTML link is brilliant and further allows easy sharing of data among laboratories and individuals. In summary, *luox* provides researchers and other professionals who desire to know the potential neuroendocrine, neurobehavioral and circadian effects of a light source a useful tool for quantifying light for these physiological effects.

Are the conclusions about the tool and its performance adequately supported by the findings presented in the article?

Yes

Is the rationale for developing the new software tool clearly explained?

Yes

Is the description of the software tool technically sound?

Yes

Are sufficient details of the code, methods and analysis (if applicable) provided to allow replication of the software development and its use by others?

Yes

Is sufficient information provided to allow interpretation of the expected output datasets and any results generated using the tool?

Yes

Reviewer Expertise:

Photobiology

We confirm that we have read this submission and believe that we have an appropriate level of expertise to confirm that it is of an acceptable scientific standard.

# Peer review: Reviewer response for version 1

**DOI:** 10.21956/wellcomeopenres.18289.r43314

**Published:** 2021-04-09

**Authors:** Timothy Brown

**Affiliations:** 1 Faculty of Biology Medicine and Health, University of Manchester, Manchester, UK

This report describes a new open source web-platform for calculating various quantities relating to light and lighting as experienced by a standard human observer. In particular, the tool facilitates calculations of metrics related to ipRGC-influenced responses to light as described in the CIE S026 international standard, as well as additional colorimetric calculations.

 There is growing appreciation that 'non-visual' effects of light can influence health and well being (e.g. via effects on the circadian system and sleep) and substantial interest in harnessing such effects for practical benefit. This tool is potentially of great utility in facilitating appropriate quantification and description of lighting conditions for scientific studies and in real-world applications. The tool also provides a simple means by which such information can be shared and referenced.

 Given that the tool is OS-independent and does not require any proprietary software this should be of great utility across the spectrum from vision/circadian scientists to lighting professionals.

 The report does not seem to directly describe cross-validation of the outputs of the web platform with existing tools (e.g. the CIE excel tool). I do not doubt the outputs of both tools are the same (and imagine the Authors have confirmed as much) but direct confirmation that this is the case would be worth including.

Are the conclusions about the tool and its performance adequately supported by the findings presented in the article?

Partly

Is the rationale for developing the new software tool clearly explained?

Yes

Is the description of the software tool technically sound?

Yes

Are sufficient details of the code, methods and analysis (if applicable) provided to allow replication of the software development and its use by others?

Yes

Is sufficient information provided to allow interpretation of the expected output datasets and any results generated using the tool?

Yes

Reviewer Expertise:

circadian and visual neuroscience

I confirm that I have read this submission and believe that I have an appropriate level of expertise to confirm that it is of an acceptable scientific standard.

# Response

**Published:** 2021-04-30

**Authors:** Manuel Spitschan

**Affiliations:** University of Oxford, UK

Thank you for your review. The cross-validation of the outputs is currently being performed by the CIE and will be written up as a report, which will be included on the website.
